# Current Status of Regulatory Non-Coding RNAs Research in the Tritryp

**DOI:** 10.3390/ncrna8040054

**Published:** 2022-07-18

**Authors:** Rafael Sebastián Fort, Santiago Chavez, Juan M. Trinidad Barnech, Carolina Oliveira-Rizzo, Pablo Smircich, José Roberto Sotelo-Silveira, María Ana Duhagon

**Affiliations:** 1 Departamento de Genómica, Instituto de Investigaciones Biológicas Clemente Estable, Montevideo 11600, Uruguay; rfort@fcien.edu.uy (R.S.F.); schavez@fcien.edu.uy (S.C.); jtrinidad@fcien.edu.uy (J.M.T.B.); psmircich@fcien.edu.uy (P.S.); 2 Laboratorio de Interacciones Moleculares, Facultad de Ciencias, Universidad de la República, Montevideo 11400, Uruguay; coliveira@fcien.edu.uy; 3 Departamento de Genética, Facultad de Medicina, Universidad de la República, Montevideo 11800, Uruguay; 4 Departamento de Biología Celular y Molecular, Facultad de Ciencias, Universidad de la República, Montevideo 11400, Uruguay

**Keywords:** trypanosomatids, tritryp, Trypanosoma, leishmania, non-coding RNA, ncRNA, tRNA, snoRNA, snRNA, siRNA

## Abstract

Trypanosomatids are protozoan parasites that cause devastating vector-borne human diseases. Gene expression regulation of these organisms depends on post-transcriptional control in responding to diverse environments while going through multiple developmental stages of their complex life cycles. In this scenario, non-coding RNAs (ncRNAs) are excellent candidates for a very efficient, quick, and economic strategy to regulate gene expression. The advent of high throughput RNA sequencing technologies show the presence and deregulation of small RNA fragments derived from canonical ncRNAs. This review seeks to depict the ncRNA landscape in trypanosomatids, focusing on the small RNA fragments derived from functional RNA molecules observed in RNA sequencing studies. Small RNA fragments derived from canonical ncRNAs (tsRNAs, snsRNAs, sdRNAs, and sdrRNAs) were identified in trypanosomatids. Some of these RNAs display changes in their levels associated with different environments and developmental stages, demanding further studies to determine their functional characterization and potential roles. Nevertheless, a comprehensive and detailed ncRNA annotation for most trypanosomatid genomes is still needed, allowing better and more extensive comparative and functional studies.

## 1. Introduction

Trypanosomatids are flagellated protozoan parasites belonging to the early branching supergroup *Discobids*, formerly part of the eukaryotic lineage of excavates, which are no longer supported as a monophyletic clade. Thus, these organisms comprise a very early divergent group in the eukaryotic evolutionary tree and show many distinctive features, which are not present in well-studied fungi, plant, or animal cells, providing numerous insights into the early establishment of cell and molecular mechanisms of eukaryotic [1,2]. Trypanosomatids belong to the class *Kinetoplastea*, named by the peculiar genome architecture of their unique mitochondrion composed of a network of circular DNA molecules called the kinetoplast [3].

Trypanosomes cause devastating vector-borne human diseases, mainly in tropical and sub-tropical regions of the world where their specific transmitting arthropods are endemic. African trypanosomiasis (sleeping sickness) caused by *Trypanosoma brucei*, American trypanosomiasis (Chagas disease) caused by *Trypanosoma cruzi*, and leishmaniasis (a set of diverse diseases) caused by various species of the genre *Leishmania*, are three major parasitoses that impact hundreds of millions of people worldwide [4,5,6]. Trypanosomatids display very complex life cycles with different developmental stages alternating between the invertebrate and the vertebrate hosts, which are usually different mammal species, thus comprising intricate zoonoses [7,8].

Several unusual gene expression mechanisms in trypanosomatids exemplify the divergence across eukaryotic organisms. First and foremost, there is no evidence of canonical core promoter elements for RNA polymerase II to transcribe the protein-coding genes. Instead, a polycistronic transcription starts bi-directionally at divergent strand-switch sites in the genome [9]. The primary polycistronic RNAs are processed into single mature mRNAs by coupled 5′ trans-splicing and 3′ polyadenylation events, leading to the removal of the intergenic regions intervening the genes [10,11]. Since most genes lack introns [12], cis-splicing is a rare event. Due to the absence of transcription initiation control, trypanosomes heavily rely on post-transcriptional events to achieve and maintain differential gene expression [13].

Following the revolution generated by the discovery of microRNAs (miRNAs) in 1993 [14] and the small interfering RNA (siRNA) pathway in 1998 [15], it became clear that non-coding RNAs (ncRNAs) comprise a very efficient, quick, and economic strategy to regulate gene expression, especially for parasite organisms that are constantly exposed to dynamic environmental and host interactions. In addition, the capacity to quantify, characterize, and annotate ncRNAs has increased exponentially with the new generation of massive sequencing technologies, leading to the discovery of new classes of ncRNAs and improving their annotation [16]. Typically, ncRNAs are arbitrarily divided by size into small and long (shorter and longer than 200 nt, respectively) [17]. However, the range of mid-sized ncRNAs (50–400 nt) has been proposed as a distinctive group based on their structures and functional features [18]. Among the small ncRNAs, we can identify: miRNAs, siRNAs, piRNAs, snoRNAs, snRNAs, tRNAs, and vtRNAs. Meanwhile, among the long ncRNAs, we can find: lincRNAs, lncRNAs, rRNAs, and telomerase RNA [16], the acronyms of which can be found in Table 1. The most studied small ncRNAs are the miRNAs and siRNAs, which are processed by Dicer and have functional roles in association with argonaute proteins (AGO). However, more recently, a new category of ncRNAs has bought the attention of the ncRNA field, namely the small RNA fragments derived from tRNAs (the most studied fragments): snoRNAs, snRNAs, and rRNA (Table 1) [19,20,21,22,23,24]. Actually, tRNA fragments and their regulatory functions have been identified across all kingdoms of life [25,26,27], pointing to the need for further studies to expand the knowledge of other ncRNA-derived fragments and their potential roles. The specific cleavage of canonical ncRNAs which produce fragments that functionally mimic miRNAs or directly interact with ribosomes, proteins (such as RNA binding proteins), or other RNAs to modulate gene expression [28,29,30]. Although these fragments were initially considered products of degradation and thus ignored in small RNA-seq analysis, the accumulated evidence supports the generation of fragments by specific processing of ncRNA [31,32,33]. However, many cases lack known processing factors or defined mechanisms of action, leaving an open field for research ahead. 

Key and unique ncRNAs were discovered very early in trypanosomes. Pioneering studies regarding editing guide RNAs [34] and splice leader RNA [35] are examples. Most of the ncRNAs classes described later for eukaryotes were found and annotated (rRNAs, tRNAs, snoRNAs, snRNAs, telomerase RNA, and vault RNA) [36,37]. Recently, novel ncRNAs antisense regulators of mRNA translation were added to the catalog [30]. Regarding small RNA fragments derived from canonical ncRNAs, most of the work is focused on tsRNAs (for review see: [38,39]); however, other fragments derived from rRNAs, snoRNAs, and snRNAs were identified [40,41]. The aim of this review is to portray the ncRNA landscape in trypanosomatids, with a special emphasis on the existence and regulatory roles of small RNA fragments derived from functional RNA molecules. 

**Table 1:** Standardized names for the ncRNAs and derived fragments.

RNA Biotype (Acronym Used)	Derived Fragments/Small RNA (Acronym Used)	Canonical Process Involved
Transfer RNA (**tRNA**)	tRNA-derived RNA fragments (**tsRNAs**) [42]	Translation
Ribosomal RNA (**rRNA**)	rRNA-derived fragments (**sdrRNA**) [40]	Translation
small nuclear RNAs (**snRNAs**)	snRNA-derived fragments (**snsRNA**) [40]	RNA processing
Small nucleolar RNAs (**snoRNAs**)	snoRNA-derived fragments (**sdRNAs**) [40]	RNA processing
Long non-coding RNAs (**lncRNAs**)	lncRNAs-derived fragments (**lncRNAs**) [43]	Gene expression
Vault RNA (**vtRNA**)	Vault RNA-derived (**svRNAs**) [44]	Vault particle
Splice leader RNA (**SL-RNA**)		RNA processing
Editing guide RNAs (**gRNAs**)		RNA Editing
Small interfering RNAs (**siRNAs**)		Gene expression
Telomerase template bearing RNA (**TR**)		Chromosome maintenance

## 2. ncRNAs Annotations in the TriTryps Genomes

A hallmark in trypanosomatid biology was set in 2005 with the release of the first full genomic sequence for the three major trypanosomes of public health relevance: *T. brucei* [45], *T. cruzi* [46], and *L. major* [47] that were eventually named as the TriTryps, and the sequenced strains were set as reference (*T. brucei* TREU 927, *T. cruzi* CL-Brener, *L. major* Friedlin). The *T. cruzi* genome of the hybrid strain CL-Brener was the largest (55 Mb and 12,000 genes) compared with the *T. brucei* genome of 26 Mb and approximately 9068 genes, and the *Leishmania* genome of 32.8 Mb with 8311 genes [48]. A comparative analysis of these genomes revealed that a core of protein-coding genes was conserved and displayed a high degree of synteny among the three species, while each organism also displayed an important set of species-specific genes that coded for functions related to the infection process [48]. Since then, a comprehensive database (TriTrypDB) was formed and has been expanded to include genomes of different strains for the TriTryps, as well as many genomic sequences for other trypanosomatid species [49]. 

**Figure 1:**
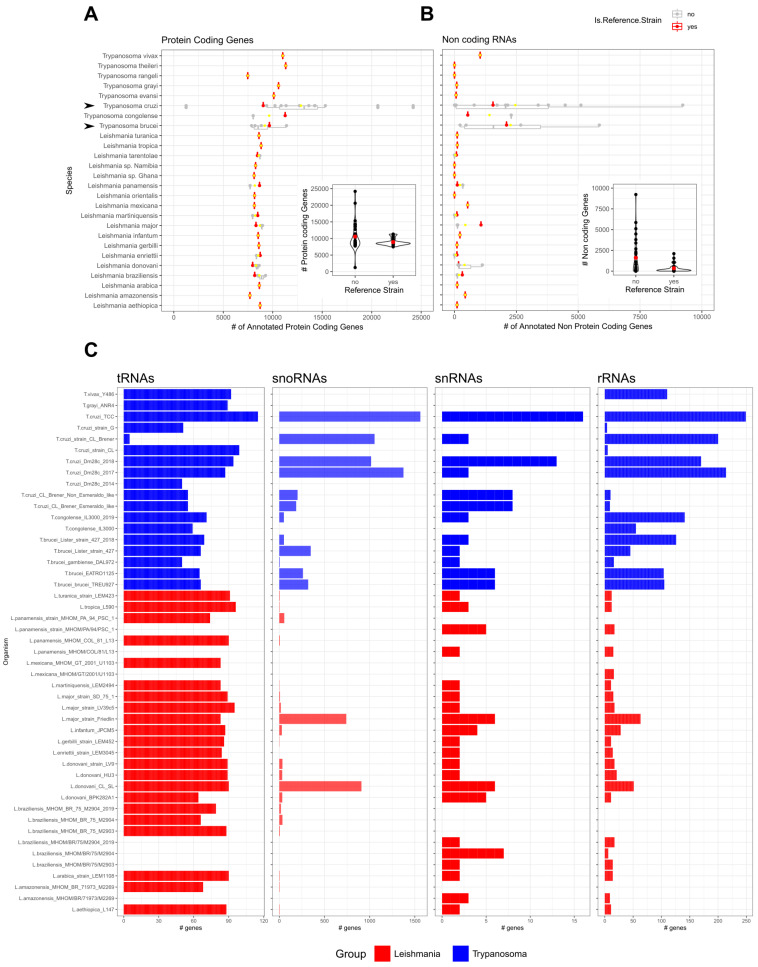
Outlook of the non-coding genomic annotations available in TriTrypDB. All *Leishmania* spp. and *Trypanosoma* spp. genomic data was obtained from TritrypDB (Release 56) for protein-coding genes (**A**) and non-protein-coding genes (**B**). The inset plot presents the distribution of the number of genes annotated for all genomes grouped by reference and non-reference strains. For the main ncRNAs biotypes (tRNAs, snoRNAs, snRNAs, and rRNAs) the number of features annotated is presented in bar plots (**C**) for all genomes with a least 1 ncRNA annotation. Arrows point out species with large numbers of genome strains sequenced.

Although at first, gene annotations were scarce and not reliable, this has been improved over the years and protein-coding genes now share more comprehensive annotations for most genomes (Figure 1A). A different scenario can be observed for the non-coding RNAs (Figure 1B) when compared with protein-coding genes (Figure 1A). Some genomes have an extensive description of protein-coding genes while lacking non-protein-coding gene annotations. In species where there are numerous strains sequenced (see arrows for *T. cruzi* and *T. brucei* in Figure 1A,B), a wide range of a total number of annotated genes can be observed both for protein-coding and non-coding. However, reference strains show less dispersion in the total number of genes (inset plots of Figure 1A,B). This could be explained by the diverse library preparation and sequencing methods utilized that finally has an impact on the genome characterization and annotations. A good example is the application of third-generation sequencing technologies which led to an improvement in the annotation of multicopy gene families that were previously collapsed and underestimated [50]. As can be seen in Figure 1C, there is not a consistent and comprehensive annotation for the ncRNAs of the main 4 biotypes (tRNAs, snoRNAs, snRNAs, rRNAs), reflected by the difference in the total number of features defined in each class for each genome. This is particularly striking for the snoRNA, snRNA, and rRNA classes. Some genomes present annotations for the four ncRNA classes, while others present annotations for only one class. The tRNA class has the most widespread annotations amongst the genomes of different parasites, while the opposite is observed for the snoRNAs class. The heterogeneity of the RNA gene annotation across species together with the scarce annotation of regulatory ncRNAs precludes direct comparisons among species. Overall, the ncRNAs landscape in trypanosomatids requires efforts on the extension and homogenization of the annotations for most genomes to allow comparative and functional studies in this expanding field.

## 3. ncRNA Biotypes

**Figure 2:**
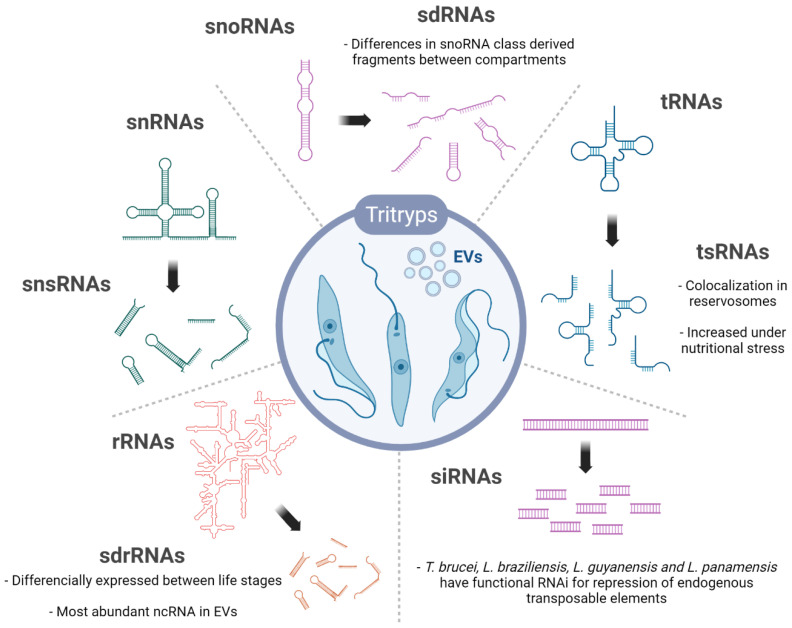
Overview of the ncRNAs and their derived sncRNAs products identified in trypanosomatids. The sncRNAs represented were found in at least one trypanosomatids, either intracellular and/or in EVs (Extracellular vesicles). snsRNA, sdRNA, tsRNA, sdrRNA are the small RNAs derived from snRNA, snoRNA, tRNA and rRNA, respectively. Specific features or observations are commented on with the text. Created with BioRender.com (accessed on 13 May 2022).

This revision focuses on the regulatory roles of trypanosomatid ncRNAs. The constitutive RNAs involved in mRNA translation (tRNA, rRNA, snoRNA) are presented because of their regulatory potential, most of which is held by the small RNAs derived from them (Figure 2). Other housekeeping RNAs, such as the SL-RNA involved in trans-splicing and the gRNAs implicated in mitochondrial RNA editing are also described due to their prominence in trypanosomatids. Further, ncRNAs are discussed because of their recent involvement in novel regulatory processes (lncRNAs, vtRNA, anti-sense RNAs).

### 3.1. tRNA and tsRNAs

Transfer RNAs (tRNAs) are one of the most abundant small non-coding RNA in the cell (5–10%), being essential components of the translation machinery delivering the amino acids to the ribosome to form polypeptides [25]. In trypanosomes, the tRNAs are transcribed by RNA polymerase III, and most of them are in clusters of 2–5 repeats in tandems (see review Shikha et al., 2019 for aspects of biogenesis and processing). Currently, the tRNA-derived RNA fragments (tsRNAs) are receiving great attention in RNA biology due to the functional implications that are revealed in all kingdoms of life [25,26,27]. The first report that revealed the presence of fragments derived from tRNA in trypanosomatids (epimastigotes of *T. cruzi*) was carried out by cloning and sequencing size selected RNAs (22 to 33 nts) [42]. Specifically, 87 clones (~26%) out of 348 correspond to tsRNAs, and ~90% of these fragments were derived from only three tRNAs isoacceptors: tRNA-Asp GUC, tRNA-Glu CUC, and tRNA-Glu UUC. These fragments were mapped to 5′ or 3′ halves of tRNAs (length ranging from 29 to 33 nt), the 5′ halves being the majority. Interestingly, FISH against some tRNA halves showed cytoplasmic granules colocalizing with TcPIWI in reservosomes [51]. Noteworthy is the exposure of late-stationary epimastigotes in increasing levels of extracellular vesicles carrying tsRNAs significantly enhanced by metacyclogenesis in axenic cultures [51]. Supporting the latter, Reifur et al., 2012, with a similar approach, showed that 63% of the cloned sequences correspond to the tsRNA of a median length of 33 nt in *T. cruzi* metacyclic forms [52]. Strikingly, in a metacyclic stage, most of the fragments (87%) are derived from the 3′ arm of the tRNAs (25% carried the 3′ CCA sequence), in contrast with the arm distribution observed in epimastigotes [42], which could imply a different processing between these two stages [52]. 

**Figure 3:**
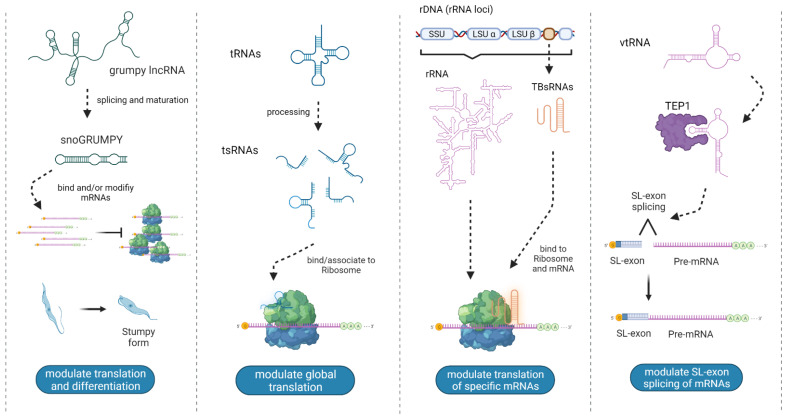
Regulatory non-canonical ncRNAs with experimentally demonstrated roles reported in trypanosomatids. The illustrations represent the structure, processing and proposed mechanisms of action of the four ncRNAs reported so far [28,30,37,57]. Created with BioRender.com (accessed on 13 May 2022).

The small non-coding RNA (sncRNA) sequencing by NGS in *T. cruzi* expanded data of tsRNA in epimastigotes, where most of the tRNAs are precursors of tsRNAs [40], specifically 89% corresponded to 3′ halves with an average length of 38 nt, and 75% of them had the CCA extension. The disagreement with the 3’ tsRNA overrepresentation previously described by Garcia-Silva et al. is likely due to the different methods used. A population of shorter fragments derived from tRNAs (<25 nt) was also identified, but at a much lower frequency [40]. A study of sncRNAs derived from extracellular vesicles (EVs) of epimastigotes and metacyclic trypomastigotes revealed large differences between the proportion of tsRNAs (average length 32 nt) identified in these compartments [53]. Another study also confirmed the presence of tRNAs halves in *T. cruzi* secreted EVs by microscopy observations using gold-labeled 5′ arm tsRNAs and demonstrated that they could be effectively delivered into mammalian cells, inducing changes in the expression levels of specific genes in mammalian cells susceptible to infection [54]. Later, a comparative analysis of sncRNAs of the intracellular and the extracellular compartments in *T. cruzi* epimastigotes under nutritional stress confirmed the presence of fragments derived from multiple ncRNAs, including tRNAs [41]. This study found that tsRNAs comprise 45% of all the fragments of EVs, but 31% in their intracellular counterparts. Notably, two major populations of fragments were observed in the intracellular fraction with median lengths of 22 and 34 nts, while a single population with a median of 34 nts was identified in EVs. In a global description of sncRNAs (<30 nt), using small RNA-seq, in *T. brucei* (slender and procyclic forms), a striking difference in length distribution of sncRNAs derived from each stage of the parasite was described [55]. Key differences were observed in tsRNAs populations, where fragments derived from tRNA-Asp were nearly half of the tsRNA reads in slender form, while in the procyclic form this particular fragment drops down to 6% [55]. Remarkably, the opposite was observed for tsRNAs derived from tRNA-Glu (18% in slender and 74% in procyclic). In the case of leishmanias (*L. donovani* and *L. braziliensis*), sncRNA-seq of axenic amastigotes exosomes revealed that the most frequent tsRNAs are derived from tRNA-Asp, tRNA-Gln, tRNA-Glu, and tRNA-Leu. Additionally, there was no correlation with the amino acid usage, as previously observed in *T. cruzi*. Regardless, the most abundant tsRNAs derived from tsRNA-Asp and tsRNA-Gln were from the 5′ arm [56]. Recently, the study of co-purified sncRNA with ribosomes in the bloodstream and procyclic *T. brucei* parasites exposed to different environmental conditions revealed many tsRNAs in the ribosome and polysome fraction, most of them 5′ halves of 33 nt [28]. Remarkably, an augmented amount of tsRNAs in stationary phase parasites (maximum in the starved parasites) compared to exponential growth was observed [28]. Additionally, the global amount and the identity of the tsRNAs observed was different among the conditions evaluated. Furthermore, by using an in vitro translation extract of *T. brucei*, 3′ tRNA-Thr halves were able to stimulate translation by at least 20%, a result that was also confirmed in transfected parasites (Figure 3) [28]. 

Overall, these observations indicate a unique tsRNA expression pattern favoring specific processing, sorting, or stabilization of tsRNAs in diverse conditions, compartments, or life stages of the parasites. Several reports show that different tsRNAs are usually synthesized/processed in normal parasite cell biology while under certain stress, which seems to be a common strategy to respond to the environmental changes. Despite tsRNAs being the most studied sncRNA fragments derived from canonical ncRNAs, further studies are needed to define the molecular mechanism and the precise relation to cell biology, stages, and pathology of the parasites. 

### 3.2. snRNA and snsRNAs 

The small nuclear RNAs (snRNAs) are highly conserved U-rich non-coding RNAs located in the nucleus, which are essential in the spliceosome complex, the machinery involved in pre-mRNA processing [58]. The major spliceosome complex which is responsible for the majority of the mRNA splicing events is formed by U1, U2, U4, U6, and U5 snRNAs. Additionally, some organisms possess a second splicing apparatus called the minor spliceosome complex (U11, U12, U4atac, U5 and U6atac snRNAs) which is involved in more specific splicing events [59,60]. The long polycistronically transcribed protein-coding genes (pre-mRNAs) of trypanosomatids are processed by coupling trans-splicing and polyadenylation to individual mRNAs by the spliceosome complex [61]. Particularly, for trans-splicing in trypanosomes, the SL RNA substitutes the function of the U1 snRNA in the complex [62]. 

Interestingly, small RNA fragments derived from specific locations of the snRNAs were found in eukaryotic model organism [19,63,64], and their possible functional role as a negative regulator of gene expression was hypothesized due to their association with AGO proteins [65,66]. In addition, snsRNAs derived from U2 snRNA were observed to increase in serum from patients with cancer [63,67]. However, more research is needed to determine snRNA-derived fragments’ role and functional mechanism in cell biology. 

In trypanosomes, small RNA-seq experiments showed fragments derived from snRNAs with a median of 40 nts. However, 82.1% of the fragments were mapped to snRNAs U4 and U5 [40]. Other studies identify small RNA fragments derived from snRNAs in *T. cruzi* [41,51,53] and *T. brucei* [28,36,55], but they no further explore other aspects related to their production, characterization, or possible regulatory function in the studies. In leishmania, the small RNA-seq of exosomal RNA derived from *L. donovani* and *L. braziliensis* found snRNA-derived fragments and identified one snsRNA in the top 20 most abundant fragments in the libraries [56].

### 3.3. snoRNAs and sdRNAs

Small nucleolar RNAs (snoRNAs) are conserved ncRNAs with a length between 60 and 250 nts, encoded in introns of host genes or controlled by independent promoters. Their main function is to serve as a guide for specific base modifications in the ribosomal RNA. Briefly, the snoRNAs are classified into two groups depending on the modification they guide on rRNA: the box C/D snoRNAs serve as a guide for 2′-O-ribose methylation, and the box H/ACA snoRNAs serve as a guide for pseudo-uridylation [68]. While the trypanosomes C/D snoRNAs group has the eukaryotic consensus structure, the H/ACA snoRNAs have only one hairpin instead of two, and carry an AGA sequence box instead of the consensus ACA motif [36]. Trypanosomes snoRNAs carry out peculiar processing and modification of rRNAs (thoroughly reviewed in [69]). Indeed, the trypanosome rRNA holds specific roles in translational regulation and cycling between hosts (see below), which is reflected in the high number of snoRNAs in trypanosomatids genomes [69,70,71]. Recently, a great number of modifications guided by snoRNAs associated with rRNA processing were described and, strikingly, some of them are developmentally regulated [72,73]. Remarkably, the overexpression of some specific snoRNAs that guide modifications in the H69 domain of the trypanosome rRNA was associated with an increasing growth rate of the parasites [30].

The discovery of stably accumulating fragments derived from snoRNAs (termed sdRNAs) led to new regulatory functions besides the known canonical main function of this molecule [19,65,74,75,76]. Furthermore, it was evidenced that sdRNAs could act like miRNAs in many model organisms: plants, protozoa, mice, and humans [66,68,74,77,78]. Recently, in addition to the miRNA-like function, it was described that levels of sdRNAs were dependent on stress conditions and may interact with translating ribosomes, promoting protein synthesis suppression in yeast [79]. In *T. cruzi*, a sncRNA sequencing experiment revealed the presence of sdRNAs with a median length of 35 nts derived from both classes of snoRNAs (C/D box and H/ACA box) [40]. Then, the RNA sequencing of snoRNA-enriched RNPs of *T. brucei* (implemented for snoRNAs gene annotation) found small RNA fragments of 20–30 nts derived from snoRNAs. Small RNA fragments were identified for 80% of C/D box and 90% of H/ACA box snoRNAs; however, the authors speculate that derived fragments could appear as the result of low-level RNA degradation during preparation and/or manipulation [36]. Posteriorly, several small RNA-seq studies supported the presence of sdRNAs in whole-cell epimastigotes, trypomastigotes, and vesicles in trypanosomatids [28,41,51,53,55]. Intriguingly, Fernandez-Calero et al. remarked that despite the similar proportion of sdRNAs fragments in EVs and their intracellular counterparts, the size distribution of reads was profoundly different between both compartments. EVs sdRNAs presented three main populations with median peaks of 26, 28, and 34 nts, while intracellular sdRNAs showed two populations with median peaks of 33 and 35 nt [41]. However, the major differences between both compartments are associated with the class of snoRNAs from which the fragments derived. The EVs fraction showed 99% of sdRNAs derived from the H/ACA box class, while most of the intracellular sdRNAs derived from the C/D box class. In Leishmania, the small RNA-seq of exosomal RNA derived from *L. donovani* and *L. braziliensis* identified a small proportion of sdRNA (~1.5%) [56]. Overall, sdRNAs are identified in trypanosomatids, revealing differences in their levels, sizes, and classes among developmental stages and different subcellular compartments. Despite these observations, the role of the sdRNAs has not been addressed in trypanosomatids yet. 

### 3.4. rRNA and sdrRNA 

Ribosomal RNAs (rRNA) are non-coding RNAs transcribed by RNA polymerase I that are indispensable components of a large RNP complex, the ribosome, essential for the translation of all proteins in the cell. Indeed, ribosomal RNA is the most abundant RNA in the cell, reaching at least 80% of the RNA mass of the cell [80]. The processing of the ribosomal RNA in trypanosomes is unique, comprising two large (LSUα and LSUβ) and four small rRNA subunit fragments (srRNA 1,2,4, and 6), with snoRNAs acting as key players in this complex process [69]. The small ribosomal RNA-derived fragments (named both sdrRNA or rRFs) are usually considered by-products of degradation and are discarded or not considered in further small RNA-seq analysis. Nevertheless, several studies have accumulated convincing evidence presenting sdrRNAs as relevant translation regulators, reviewed in [21]. Certainly, the canonical rRNA processing involves different nucleases that might produce small RNA fragments during rRNA maturation that could eventually be functional molecules [19,81]. The systematic study of small RNA sequencing datasets found an extensive presence of sdrRNAs and demonstrated that these are mainly mapped to the rRNA coding regions in the sense orientation (64–70% mapped to large 28S rRNA subunit [82]). Furthermore, the sdrRNAs were found co-immunoprecipitated with Argonaute (AGO) proteins [65,66,82]. Remarkably, the functional role as translation regulators of some sdrRNAs was demonstrated in Neurospora [83] and human models [84]. In fact, Chen et al. demonstrated that RNAi knockout of one of the selected sdrRNAs induces apoptosis and inhibits cell proliferation in H1299 human cells [84]. More recently, meta-analysis of CLASH and AGO-PARCLIP datasets indicates that sdrRNAs are non-randomly generated and confirms their association with AGO proteins [24]. Indeed, this analysis led the authors to identify multiple non-randomly generated pairs of sdrRNAs and cellular transcripts, revealing several motifs of double-stranded binding regions. This observation led the authors to hypothesize a possible mechanism of unwinding mRNAs to regulate their translation [24]. 

In trypanosomes, a small RNA sequencing experiment of epimastigotes of *T. cruzi* revealed that 17% of reads derived from rRNA and showed a three-peak length distribution of 20, 33, and 46 nt [40]. Similarly, in a small RNA-seq study on infective metacyclic forms of *T. cruzi*, 25% of reads mapped to rRNAs [52]. Remarkably, Zheng et al. found sdrRNAs as one of the most differentially expressed sncRNAs (most of them ~20 nt length) in slender versus procyclic forms of *T. brucei* [55]. Furthermore, one of the studied sdrRNAs, was derived from a hairpin structure at the 3′ end of the 28S rRNA and was present in both stages but significantly increased in the procyclic form (14% of all reads). Furthermore, the authors speculate about a possible functional relevance of this sdrRNA based on the high conservation of its sequence among eight genetically related species [55]. Posteriorly, the small RNA sequencing analysis of RNAs associated with TcPIWI (an AGO/PIWI protein) of *T. cruzi* epimastigotes revealed that most of the sncRNAs (95%) identified were fragments derived from rRNA [85]. Then, the characterization of the small RNAs of EVs from two stages of *T. cruzi* (epimastigotes and metacyclic trypomastigotes) determined that the most abundant class in all samples were the sdrRNAs, supporting previous observations [51,53]. Remarkably, Evs secreted by *T. cruzi* (carrying large amounts of sdrRNAs) provoked changes in the gene expression level of many genes in host HeLa cells upon its incorporation [54]. Indeed, it was observed that *T. cruzi* trypomastigotes display a 5-fold increase in invasion over cells pre-incubated with trypomastigotes Evs [86]. The comparative analysis of EVs and their intracellular counterpart from *T. cruzi* epimastigotes under nutritional stress revealed that sdrRNAs were the most abundant class of small RNAs, being 46% and 58%, respectively [41]. However, the length of this sdrRNA was different between both compartments; intracellular distribution showed peaks at 19, 29, and 36 nt, while the 29 nt peak was not observed in EVs. Similar to previous observations, it was determined that sdrRNAs derive from specific locations of the rRNA genes, supporting the idea of site-specific processing rather than random degradation [41]. Additionally, some of these sdrRNAs seem to be differentially enriched between compartments, which could be explained by their specific processing or sorting/packaging [41]. Recently, sdrRNAs were observed in the small RNA interactome of ribosomes in bloodstream and procyclic *T. brucei* parasites exposed to different environmental conditions [28]. These fragments were the most abundant class of small RNAs in almost all the conditions tested, except for parasites submitted to starvation, where the tsRNA reached its maximum and sdrRNAs reached their minimum. In Leishmania, the small RNA sequencing of exosomes derived from early axenic amastigotes of *L. donovani* and *L. braziliensis* which revealed a 31% and 15% of reads mapping to rRNAs, respectively [56]. Remarkably, the peak of the size distribution of these sdrRNAs was different between *L. donovani* (39 nts) and *L. braziliensis* (52 nts). In contrast with previous observations, site-specific enrichments could not be determined for the fragments derived from rRNA genes, and 90% of the mappings were distributed in both 28S and 18S rRNA genes [56]. It is worth noting that the small RNA library preparation for this study involves CIP (Calf Intestinal Phosphatase) and TAP (tobacco acid pyrophosphatase) treatment, which would incorporate RNA fragments of different 5′-end nature which could explain the discrepancies mentioned. In addition, new evidence suggests that rRNA genes are also a source of larger (90–500 nt) functional ncRNAs in *T. brucei* [30]. The later study applied RNA sequencing on the post-ribosomal supernatant revealing the presence of novel developmentally-regulated ncRNAs [30]. These ncRNAs are located and processed from the pre-rRNAs’ internal transcribed spacer (ITS) and external transcribed regions (ETS). Remarkably, by using a UV cross-linking-ligation approach, it was described that these ncRNAs could interact (through base pairing) and move together with ribosomes to the cytoplasm, where they can interact and modulate the translation of a specific subset of mRNAs, evidenced by reporter and western blot assay [30] (Figure 3). Overall, ncRNAs derived from rRNAs can be observed in RNA-seq datasets in several trypanosomatids. Some fragments displayed differential expression over different developmental stages, subcellular compartments, or growth conditions, but these observations were not complemented with a functional approach in trypanosomatids. 

### 3.5. siRNAs

In eukaryotes, RNA interference pathway (RNAi) is broadly conserved, playing key roles in defense against invading viruses, regulation of gene expression, programmed DNA rearrangements, and genome surveillance [87,88]. The RNAi pathway in protist, described for the first time in 1998 in a trypanosomatid [89], is largely dedicated to siRNA. Indeed, since the canonical miRNA biogenesis pathway is not present in Protista, neither precursors and mature miRNAs nor the microprocessor machinery (Drosha, Pasha) exist in trypanosomatids [90].

In the Trypanosoma genus, only *T. brucei* have functional RNAi, which involves a canonical AGO, two Dicers (one nuclear and one cytoplasmic), and two so-called RNA Interference Factors [91,92,93,94,95]. The main role of this system is the repression of endogenous transposable elements through 24–26 nt siRNAs (TbsiRNAs), most of which are derived from INGI and SLACS elements (~78%) [55,94,96,97]. Additional TbsiRNAs derived from pseudogenes formed between antisense pseudogene and their cognate coding genes (mostly VSG) or pseudogene–pseudogene pairs (mostly RHS), lead to a TbDCL1 (cytoplasmic Dicer) dependent gene repression [98]. Furthermore, TbDCL2 (nuclear Dicer) dependent TbsiRNA (21–27 nt) are known to derive from convergent polycistronic transcription units; yet, no associated gene regulatory activity is currently proven [97,99]. Among Leishmania genus, only the species from the early diverging Viannia subgenera (*L. braziliensis*, *L. guyanensis*, *L. panamensis*) have functional RNAi pathways comprising orthologues of AGO and Dicer genes [100,101]. The 20–25 nt siRNAs of *L. braziliensis* are mainly derived from SLACS and TATE transposable elements (~75%) [102]. Remarkably, these siRNAs were found in exosomes from *L. braziliensis*, and thus they might be involved in intercellular communication [56]. Overall, the *T. brucei* and *L. braziliensis* RNAi pathways show organism-specific diversifications with implications for the production of dsRNA and siRNA [102]. The size of the siRNA fragments, the modification of the 3´end, and the addition of non-coded Us varies among the groups. The exogenous manipulation of siRNA has quickly become an approach to downregulating gene expression in the RNAi competent trypanosomatids.

Strikingly, the siRNA pathway became non-functional in the remaining trypanosomatids [100,101]. The Leishmania subgenera (*L. major*, *L. donovani*, *L. mexicata* and *L. tarentolae*) contains only remnant, highly degenerated AGO1 pseudogenes, or lack one or more of the RNAi pathway genes [100,101]. Three evolutionary mechanisms have been proposed as drivers of the RNAi loss: avoidance of dsRNA viral infections strongly relying on the RNAi pathway, loss of mobile genetic elements make the RNAi less necessary, and direct phenotypic selection since this loss affects gene expression [100]. It is worth mentioning that the bilaterian host defense system for transposon silencing, known as the piRNA pathway, is absent in trypanosomatids, and so are the piwi-associated RNAs (piRNAs) [103].

### 3.6. vtRNA

Vault RNAs (vtRNAs) are a class of eukaryotic non-coding RNAs of generally 84 to 140 nt, transcribed by the RNA polymerase III, firstly identified for its association with the vault particle [104,105,106]. Although this particle is the largest known cellular ribonucleoprotein complex, its function remains unclear. Intriguingly, as seen in studies in vertebrates, only 5–20% of the total vtRNA transcripts are associated with the vault particle, and therefore, vtRNAs probably have additional independent roles [107,108,109].

The first evidence of the presence of vtRNA in trypanosomatids comes from high-throughput sequencing of small RNAs *T. brucei* [36]. The vtRNA identified here, TBsRNA-10, is more abundant in the bloodstream than the procyclic form parasites and is mostly localized in a non-nucleolar single focus in the nucleus. It was not until 2019 that TBsRNA-10 was proposed as a vtRNA in *T. brucei*, based on secondary structure prediction and sequence similarities of short stretches in the 5′ and 3′ regions that form the bulged terminal helix [37]. Furthermore, they demonstrated that TBsRNA-10 is transcribed by pol III and is very efficiently co-immunoprecipitated with a candidate TEP1 protein, the protein that associates with mammalian vtRNAs and is required for the vault particle assembly. The authors also looked for additional examples of vtRNA genes in trypanosomatids genomes and identified candidate vtRNA genes in all searched trypanosomatid species [37]. The expression of the *L. braziliensis* vtRNA of approximately 190 nt was validated and represents the longest known member of the vtRNA family in eukaryotes. Interestingly, it was demonstrated that the silencing of the vtRNA in the bloodstream form of *T. brucei* resulted in decreased production of the SL exon-enriched long RNA products (Figure 3). Since the only TROVE containing protein identified in trypanosomatids is TEP1, and no Ro protein or YRNAs has been found in their genomes and transcriptomes, it appears that the vtRNA holds a Y RNA-like function in RNA metabolism [37].

Overall, the recent annotation of vtRNAs opens the opportunity to reanalyze trypanosomatid RNA-seq datasets to study/describe patterns of expression for these ncRNAs. Furthermore, in other organisms, the vtRNAs were identified as precursors of derived sncRNAs (svRNAs) with miRNA-like functions but generated by a different pathway from canonical miRNAs biogenesis [44,110,111,112]. The presence of these svRNAs has not been addressed in trypanosomatids yet.

### 3.7. Editing gRNAs

RNA editing was first observed in trypanosomes when the insertion of four non-DNA encoded uridine nucleotides was described to restore the coding capacity of the mitochondrial cytochrome C oxidase subunit 2 gene, which presents a frameshift in the genomic sequence [113]. Later, uridine insertions and deletions were found to be frequent and abundant for mitochondrial mRNAs, and this process was confirmed as a post-transcriptional modification on the primary RNAs [114,115,116]. Nowadays, the term RNA editing is used to describe processes in which the sequence information present in an RNA molecule is altered post-transcriptionally, and it can be classified into two general types of events: substitution and insertion/deletion, both affecting all major cellular RNAs (mRNA, rRNA, tRNA) and described widely among eukaryotes [117]. 

Trypanosomes are distinguished by the presence of a single mitochondrion containing a kinetoplast nucleoid composed of a bipartite mitochondrial genome and histone-like basic proteins. The kinetoplast DNA (kDNA) consists of a densely packed network of interlinked circular DNA molecules of two kinds, referred to as maxicircles and minicircles. Maxicircles are equivalent to the mitochondrial genome in most organisms, thus encoding ribosomal RNAs, two ribosomal proteins, and 16 respiratory-related genes, mostly cryptogenes, meaning they need to be edited in order to restore a protein-coding capacity. This editing is abundant as there can be hundreds of editions for each mitochondrial mRNA. The edition is directed by hundreds of guide-RNAs mostly encoded by the multicopy (102–103) ~1 kb DNA molecules named minicircles [118]. 

Editing guide RNAs (gRNAs) are small RNAs (50–60 nt) with a 5′ triphosphate and a 3′ oligo-U tail [119]. T3/T7-like RNA polymerases bidirectionally transcribes these RNAs to generate overlapping sense and antisense precursors. Then, these precursors undergo three sequential and well-studied 3′-end processing events involving degradation and uridylation in the mitochondrial 3′ proccesome (MPsome). These events lead to a mature U-tailed sense gRNA that can be incorporated into the editing complex (RESC, RNA-editing substrate-binding complex) and proceed to the editing pathway [120,121]. The model for edition mediated by gRNAs was described based on the perfect sequence complementarity between the gRNA and the mature mRNAs within the edited region [34]. Thus, guide RNAs specify the position and the number of uridines inserted or deleted by hybridizing to pre-mRNA and forming a series of mismatches [122,123]. First, an initial interaction between the gRNA and the pre-edited mRNA is established through a short (10–12 nt) region of complementarity, referred to as the gRNA’s 5′ anchor region. An imperfect duplex is then formed with pre-mRNA and the remaining gRNA sequence that can result in a loop out of single-stranded uridines in mRNA (then a deletion site) or a loop of purine nucleotides in gRNA (forming an insertion site). Later, the first unpaired nucleotide in the mRNA next to the 5′ anchor duplex is cleaved, triggering either a 3′-5′ degradation for the uridines exposed in the mRNA, resulting in a deletion event, or the filling of the single-stranded gap in the duplex, resulting in an insertion event [123].

The nearly complete repertoire of gRNA molecules was determined by RNA sequencing for the procyclic stage of *T. brucei* [124], describing 642 major sequence classes. Later, the bloodstream from gRNA transcriptome was described using the same protocol [125], and it was noted that RNA editing was developmentally regulated in *T. brucei*. A different approach was carried out in *L. Tarentolae* [126], using the PacBio platform to provide unambiguous complete kDNA minicircle sequences. Then, two size-selected mitochondrial-enriched RNA libraries (small under 200 nt, and large over 200 nt) were mapped to the minicircles sequences to verify the transcription of the gRNA genes. This study revealed that both libraries display mappings over both strands of the entire minicircle kDNA, suggesting that transcription is bidirectional and involves almost the full minicircle sequence, which eventually is processed to produce the mature gRNAs molecules. gRNAs comprise a relatively abundant small ncRNA in trypanosomatids that should stay enclosed in the mitochondria. In this context, non-canonical regulatory functions are less likely to emerge and be reported for these molecules.

### 3.8. Splice Leader RNA

Trans-splicing involves the precise joining of two distinct RNA molecules, and this spliceosomal processing was firstly described in *T. brucei* when a common 39-nucleotide sequence, named spliced leader (SL), was found at the 5′-end of different variant surface glycoprotein transcripts [35,127]. Later, trans-splicing was involved in the processing all protein-coding genes in trypanosomatids [128], while traditional cis-splicing only takes place in a couple of genes [12,129]. Since trypanosomatid protein-coding genes are transcribed into large polycistronic RNAs, mRNA maturation differs from most eukaryotes [9]. In this scenario, the SL addition coupled with polyadenylation dissects the polycistronic transcripts while providing the unusual cap (namely, cap4) to the mature mRNAs [130]. The leader sequence comes from a small RNA (SL-RNA) of approximately 140 (141 nt for in *T. brucei*) with a short half-life. The SL-RNA is transcribed from multiple loci in the genome, being this gene is the only one under the control of conventional RNA polymerase II promoter sites in trypanosomes [131,132]. Up to now, there are no extra regulatory functions or modifications for the SL-RNA, despite being one of the more abundantly produced RNAs in trypanosomes. 

### 3.9. Telomerase RNA 

Since the initial discovery of telomerase as the solution to the end-replication problem of eukaryotic chromosomes, much progress has been made in the identification of telomerase core components: the catalytic telomerase reverse transcriptase (TERT) and intrinsic template-bearing telomerase RNA (TR) [133]. From the TERT/TR complex, the TERT domain structures are conserved across different species, but the TR molecules vary greatly in sequence, size, and structure (from ~150 nt in ciliates to ~1.800 nt in *P. falciparum* [134]. 

Besides common features, *T. brucei* TR (TbTR) has several unique attributes that suggest a mechanistically different process of telomere maintenance [135]. In *T. brucei,* the TbTR of 900 nt RNA (longer than vertebrates TR) is encoded by a single-copy gene transcribed by pol II [134,136]. Despite their length, the TbTR presents a unique ciliate-like shorter stem-loop at the 3´ end of the template instead of a typical PK domain [133,134]. In addition, instead of the H/ACA box domain higher eukaryotes TRs, the TbTR presents a consensus sequence of snoRNA C/D box, also present in other kinetoplastids [135,136,137]. This C/D box is generated between the 5´ and 3´ ends of the TbTR and has been shown to bind the C/D snoRNA core protein NOP58 [136]. Recent evidence revealed stage differences in the TbTR structure that influences telomerase activity and consistent population doubling and parasite infection [138]. Since the TR sequence is variable among species, it is interesting to unravel whether TR has non-canonical functions in kinetoplastids and how TR sequence differences affect them. It is worth noting that in model organisms, TR non-canonical functions have been discovered, including a role in developmental myelopoiesis, cell fitness and apoptosis [139,140]. 

### 3.10. lncRNAs 

Long non-coding RNAs (lncRNAs) are ncRNAs longer than 200 nt that do not code for functional proteins. Several thousand of this RNA biotype are transcribed by pol II in eukaryotic genomes; as an example, the human genome may contain more than 100.000 lncRNA genes [141]. Even though there is debate about the biological function of most lncRNA, the relevance of several of these molecules is well established in the literature (see [142] for a recent review). In trypanosomatids, these molecules were initially described by Kolev et al. in one of the first reports of using RNA-seq in *T. brucei* [143]. However, evidence of a functional role in these organisms has only recently emerged. Studies estimate several hundred lncRNAs [57,144], mostly present in previously unannotated regions of the *T. brucei* genome. Only a few have been experimentally characterized with proposed roles in translation regulation [30] parasite development and present a variable expression in culture conditions compared to wild type cells [57,144]. Interestingly, the work by Guegan et al. shows that a lncRNA in T. brucei plays a key role in promoting differentiation to the stumpy form. This effect is mediated by the processing of the lncRNA into a snoRNA, snoGRUMPY, which regulates translation of its mRNA targets [57] (Figure 3). This work points to the significance of this biotype in trypanosomatid biology, a subject that has been mostly unexplored in this group of parasites. 

## 4. Conclusions and Perspectives

The complex trypanosomatid ncRNA landscape displays the presence of both eukaryotic canonical ncRNA and some abundant family-specific ncRNA (SL-RNA and editing gRNA) (Table 1). However, as was shown in Figure 1, a comprehensive and detailed ncRNA annotation for most trypanosomatid genomes is still needed, which will allow for better and more extensive comparative and functional studies. The advent of high throughput RNA-sequencing technologies reveals the presence of small RNA fragments derived from canonical ncRNA and the discovery of new developmentally regulated ncRNAs. Nevertheless, the different RNA-seq studies show some discordant observations that could rely on actual biological differences or be associated with the different library and sequencing approaches used (size-selection, RNA 5′/3′-end modifications, and sequencing technologies) (Table 2). These experimental designs naturally impact the RNA subsetting included in the finally sequenced library. Much effort is currently leading to developing new strategies to reduce biases associated with library and sequencing approaches, and to capture a broader range of ncRNAs present in the cell. 

Small RNA fragments derived from several canonical ncRNAs (tsRNAs, snsRNAs, sdRNAs, and sdrRNAs) were identified as the most commonly studied trypanosomatids, displaying changes in their levels among developmental stages and diverse growing conditions, as well as their distribution over subcellular compartments. These observations demand further studies for characterization and functional analysis with special emphasis on defining possible mechanisms of action. The latter could expose new layers of post-transcriptional gene expression regulation, a crucial level of control in the biology of these parasites. Furthermore, considering that in trypanosomatids the miRNA pathway is absent, while the siRNA pathway is present only in some lineages of the phylogenetic tree, functional characterization may be challenging, but could offer interesting new insights into the mechanism of action.

## Figures and Tables

**Table 2:** Small RNA transcriptomic studies in Trypanosomes. RNA-sequencing studies that quantify endogenous small RNA fragments (produced without fragmentation steps in the library protocol) are included. RNA biotypes identified in the manuscript are listed and the mainly focused ones are highlighted in bold letters. The 5´/3´-RNA end modifications described in the library preparation are presented in parenthesis. *PNK*—T4 Polynucleotide Kinase, *CIP*—Calf Intestinal Phosphatase, *TAP*—tobacco acid pyrophosphatase.

Organism	RNA Biotypes Studies	Sample Origin	RNA Size Selection	Library Type	Sequencing Technology	Reference
*T. brucei*	**siRNAs**	High-speed pellet and supernatant fractions	20–30 nt	Cloned	Sanger sequencing	[96]
*L. tarentolae*	**gRNAs**	Kinetoplast RNA	15–600 nt	5′/3′RACE	Sanger sequencing	[145]
*T. cruzi*	**tRNAs**	Epimastigotes normal growth conditions or under nutritional stress	18–40 nt	Cloned	Sanger sequencing	[146]
*T. brucei*	**siRNAs**	Bloodstream	18–30-nt	Ligation and cDNA	Illumina sequencing	[98]
*T. cruzi*	**tRNAs**, rRNAs, snRNAs and snoRNAs	Epimastigotes	16–60 nt	Ligation and cDNA	454 sequencing	[40]
*T. brucei*	tRNAs, rRNAs, snRNAs and **snoRNAs**	Procyclic small RNPs complexes	20–30 nt	Ligation and cDNA	Illumina sequencing	[36]
*T. cruzi*	**tRNAs**	Metacyclic trypomastigotes	18–40 nt	Cloned	Sanger sequencing	[52]
*T. brucei*	**siRNAs**	Epimastigotes TbAGO1 immunoprecipitates mut Dicer 1/2	18–30 nt	Ligation and cDNA	Illumina sequencing	[97]
*L. braziliensis*	**siRNAs**	Promastigotes RNA immunoprecipitated with BB2-LbrAGO1	18–30 nt	Ligation and cDNA	Illumina sequencing	[102]
*T. brucei*	**siRNAs**, **tRNAs**, **rRNAs**, snRNAs and snoRNAs	Slender form and procyclic form	18–30 nt	Ligation and cDNA	Illumina sequencing	[55]
*T. cruzi*	**tRNAs**, rRNAs, snRNAs and snoRNAs	Epimastigotes TcPIWI-tryp bound sRNAs	16–50 nt	Ligation and cDNA	Illumina sequencing	[85]
*T. cruzi*	**siRNAs**, **tRNAs**, **rRNAs**, snRNAs and snoRNAs	EVs/parental cells from epimastigotes and metacyclic trypomastigotes	16–40 nt	Ligation and cDNA	Illumina sequencing	[53]
*T. brucei*	**gRNA**	Procyclic form enriched mitochondrial vesicles	40–80 nt	Ligation and cDNA (*PNK treatment*)	Illumina sequencing	[124]
*L. donovani and L. braziliensis*	**siRNAs**, **tRNAs**, rRNAs, snRNAs and snoRNAs	Exosomes from early axenic amastigotes	20–250 nt	Ligation and cDNA (*CIP and TAP treatment*)	Illumina sequencing	[56]
*L. tarentolae*	**gRNA**	Purified kinetoplast mitochondrial	<200 nt	Ligation and cDNA (*PNK treatment*)	Illumina sequencing	[126]
*T. cruzi*	**tRNAs, rRNAs, snRNAs and snoRNAs**	Epimastigotes EVs and intracellular	<60 nt	Ligation and cDNA	Illumina sequencing	[41]
*T. brucei*	**gRNA**	Bloodstream form enriched mitochondrial vesicles	40–80 nt	Ligation and cDNA (*PNK treatment*)	Illumina sequencing	[125]
*T. brucei*	**siRNAs**	Epimastigotes WT and H3.VKO	18–30 nt	Ligation and cDNA	Illumina sequencing	[99]
*T. brucei*	**tRNAs**, rRNAs, snRNAs and snoRNAs	RNAs co-purify with cytosolic ribosomes of parasites grown under different conditions	20–300 nt	Ligation and cDNA	Illumina sequencing	[28]
